# BACE-1 inhibition prevents the γ-secretase inhibitor evoked Aβ rise in human neuroblastoma SH-SY5Y cells

**DOI:** 10.1186/1423-0127-18-76

**Published:** 2011-10-21

**Authors:** Anne Jämsä, Oscar Belda, Michael Edlund, Erik Lindström

**Affiliations:** 1 Medivir AB, Lunastigen 7, PO Box 1086, S-14122 Huddinge, Sweden

## Abstract

**Background:**

Accumulation of amyloid β-peptide (Aβ) in the plaques is one of the major pathological features in Alzheimer's disease (AD). Sequential cleavage of amyloid precursor protein (APP) by β-site APP cleaving enzyme 1 (BACE-1) and γ-secretase results in the formation of Aβ peptides. Preventing Aβ formation is believed to attenuate AD progression and BACE-1 and γ-secretase are thus attractive targets for AD drug development.

**Methods:**

Combining BACE-1 and γ-secretase inhibition on Aβ secretion from human neuroblastoma SH-SY5Y cells was evaluated in this study. Secreted Aβ40 and Aβ42 levels were measured from SH-SY5Y cells stably transfected with APPwt or APPswe genes. A selective BACE inhibitor and the γ-secretase inhibitor LY450139 (semagacestat) were used to inhibit respective secretase.

**Results:**

LY450139 increased Aβ40 and Aβ42 secretion from SH-SY5Y APPwt cells at low concentrations (by 60% at 3 nM) followed by subsequent inhibition at higher concentrations (IC_50 _90 nM). Washout studies showed that the Aβ increase evoked by 3 nM LY450139 was not due to enhanced cleavage following substrate accumulation but rather to activation of Aβ formation. By contrast, LY450139 inhibited Aβ formation from SH-SY5Y APPswe in a monophasic manner (IC_50 _18 nM). The BACE inhibitor *per se *inhibited Aβ secretion from both SH-SY5Y APPwt and SH-SY5Y APPswe cells with IC_50_s ranging between 7 - 18 nM and also prevented the increased Aβ secretion evoked by 3 nM LY450139. Combining the BACE inhibitor with higher inhibitory concentrations of LY450139 failed to demonstrate any clear additive or synergistic effects.

**Conclusion:**

BACE-1 inhibition attenuates the Aβ increase evoked by LY450139 while not providing any obvious synergistic effects on LY450139-mediated inhibition.

## Background

Alzheimer's disease (AD) is the most common form of dementia in human with amyloid plaques and neurofibrillary tangles being hallmark features. The enzymatic cascade involved in the formation of Aβ1-40 and Aβ1-42 peptides, the predominant species of plaques, has been characterized in detail (for a recent review see [1]). Amyloid precursor protein (APP) is cleaved by β-site APP cleaving enzyme-1 (BACE-1) followed by subsequent cleavage by the γ-secretase complex to form Aβ peptides. It is still not clear what the assumed neurotoxic agent is, although recent data suggest Aβ dimers and oligomers as being the most neurotoxic Aβ assemblies [2].

Nonetheless, it is widely believed that inhibiting the formation of Aβ, either by inhibiting BACE-1 or γ-secretase would be of benefit for AD patients, regardless which Aβ assembly is the neurotoxic agent. Quite some progress has been made with respect to γ-secretase inhibition. The furthest advanced compound LY450139 (semagacestat) was shown to lower Aβ levels in the cerebrospinal fluid from healthy volunteers [3]. Other γ-secretase inhibitors have achieved similar results clinically. Hence, central efficacy appears clinically feasible with this class of drugs. However, safety issues have been raised by inhibiting this drug target since γ-secretase also cleaves Notch protein, a substrate that plays an important role in cellular differentiation. Indeed, γ-secretase inhibitors have produced hyperplasia of intestinal Goblet cells and altered tissue morphology in rodents [4,5]. Also, inhibitors cause thymus atrophy preclinically [5] and reduce circulating B cells in patients [6]. Two subsequent γ-secretase inhibitors, begacestat [7] and BMS708163 [8], with improved selectivity towards Notch have reached clinical development. Nonetheless, Notch liabilities may limit the doses that can be given safely.

Inhibition of γ-secretase leads to Aβ reductions in plasma and in brain if desired compound levels are reached. Interestingly, after lowering Aβ levels at efficacious doses, Aβ subsequently rise to levels substantially higher than baseline levels, often referred to as a rebound effect. However, low, sub-efficacious doses of γ-secretase inhibitor also appear to increase Aβ levels putting the mechanism behind the rebound phenomena into question and instead suggesting an Aβ rise at low concentrations without previous inhibition. This Aβ rise phenomena has mainly been demonstrated in plasma in mice, guinea pigs, beagle dogs and healthy human volunteers [9-11] but also in cerebrospinal fluid in guinea pigs [10] and in rat brains [12]. The Aβ rebound/rise phenomena seems to be a target class-related effect, since similar findings have been demonstrated with chemically distinct γ-secretase inhibitors [7]. However, a recently characterized γ-secretase inhibitor, PF-3084014 appears to lack this attribute preclinically [13]. The possible impact of Aβ rebound/rise on AD disease progression is unknown; however it is not inconceivable that non-compliant patients could be exposed to sub-efficacious levels of γ-secretase inhibitor resulting in elevated concentrations of Aβ. Recently, a phase III clinical trial with LY450139 (semagacestat) in AD patients was discontinued prematurely [14]. Surprisingly it was reported that patients receiving LY450139 fared worse than placebo-treated controls with respect to cognitive symptoms.

Less progress has been made with respect to BACE-1 inhibition. Although BACE inhibitors reduce Aβ levels in brain or cerebrospinal fluid in PgP KO mice [15], APP transgenic mice [16-18], wild type mice [19] and rhesus monkey [20], there is limited data demonstrating central Aβ-lowering effects in man. The lack of progress of clinical BACE inhibitors is due to the difficulty of combining adequate potency with good PK properties (e.g. permeability over the BBB, efflux, protein binding, metabolism).

Considering that 1) γ-secretase inhibitors have possible safety issues which may reduce the doses regarded as safe and 2) most γ-secretase inhibitors appear to cause increases of Aβ levels at low concentrations and 3) central efficacy with BACE inhibitors is difficult to achieve, the aim of the present study was to evaluate if BACE-1 inhibition could prevent the Aβ rebound/rise evoked by a γ-secretase inhibitor and if synergistic efficacy on Aβ secretion could be achieved by combining BACE and γ-secretase inhibitors.

## Materials and methods

### Inhibitors

**Figure 1:**
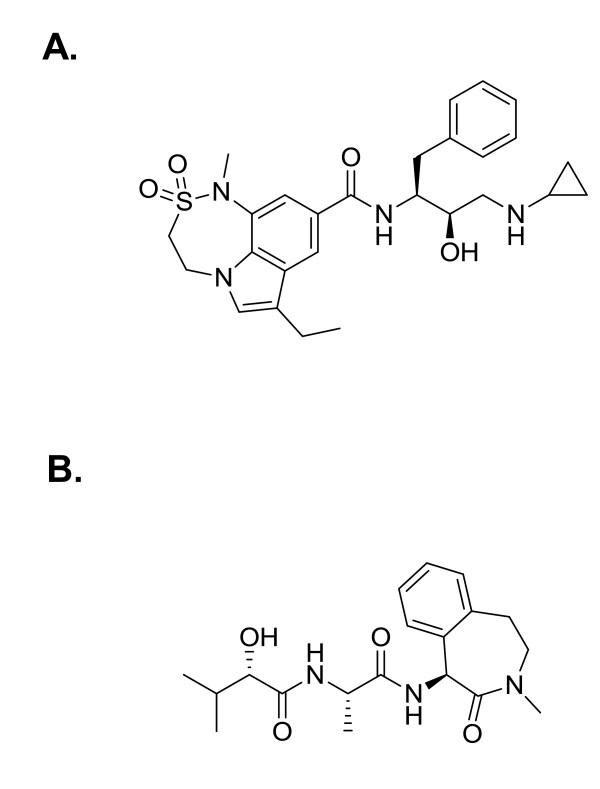
**Molecular structures of the inhibitors**. **A**. Molecular structure of the BACE inhibitor as described in Charrier et al., 2008 [22]. **B**. Structure of the γ-secretase inhibitor LY450139 (semagacestat, Audia et al., 2007 [21]).

The γ-secretase inhibitor (N)-((*S*)-2-hydroxy-3-methyl-butyryl)-1-(L-alaninyl)-(*S*)-amino-3-methyl-2,3,4,5-tetrahydro-1H-3-benzazepin-2-one dihydrate (LY450139, semagacestat) was made in-house as described in Audia et al. [21]. The BACE-inhibitor N-[(1*S*,2*R*)-1-Benzyl-3-(cyclopropylamino)-2-hydroxypropyl]-7-ethyl-1-methyl-3,4-dihydro-1H-[1,2,5]thiadiazepino[3,4,5-hi-]indole-9-carboxamide 2,2-Dioxide (compound 8e in Charrier et al., 2008) was made in-house as described in Charrier, et al. [22]. The compound is referred to as "BACE inhibitor" throughout the present paper. The molecular structures of the BACE inhibitor and LY450139 are shown in Figure 1.

### Cell culture

The human neuroblastoma cell line SH-SY5Y was purchased from European Collection of Cell Cultures (ECACC). Cells were grown in DMEM/Ham's F-12 (PAA), supplemented with 1% non-essential amino acids (PAA) and 10% Fetal Bovine Serum (PAA). Cells were maintained at 37°C in a humidified atmosphere containing 5% CO_2_.

SH-SY5Y cells were stably transfected with plasmids carrying human APPwt or APPswe gene. APP gene was purchased from Geneservice Ltd (clone sequence BC065529; clone MGC75167) and the APPswe mutation was generated using a QuikChange^® ^Site-Directed Mutagenesis Kit (Stratagene). APPwt and APPswe genes were cloned into a mammalian expression vector pcDNA3.1 and the expression was under the control of CMV promotor. The cells were plated at the density of 6.25 × 10^4 ^cells/cm^2 ^in 6-well culture dishes (Sarstedt) and each well was transfected with 4 μg plasmid DNA. Lipofectamine™2000 (InVitrogen) was used as a transfection reagent according to manufacturer's instructions. The transfected cells were selected with 400 μg/ml G418 (PAA).

Cells were plated at a density of 6.25 × 10^4 ^cells/cm^2 ^in 96-well cell culture plates (TRP) for Aβ measurements with ELISA or in 6-well culture dishes (Sarstedt) for Western blot. The day after the plating of the cells, the cultures were treated with various concentrations of γ-secretase inhibitor LY450139 or the BACE inhibitor for 24 hours. Stock solutions of inhibitors were prepared in dimethyl-sulfoxide (DMSO). All cultures including the control cells received equal amounts of DMSO, the final concentration being 0.1%. All the results were from two to three separate experiments and the data presented as means ± SEM.

### ELISA

The secreted Aβ40 and Aβ42 peptides in cell culture media were measured using human amyloid β40 or β42 ELISA kits from Millipore. The absorbance was measured at 450 nm by Microplate reader (Molecular Devices). Data from inhibitor-treated cell cultures was expressed as a percent of untreated controls and the inhibition curves were analysed by non-linear regression using Graph Pad Prism.

### Western blot

SH-SY5Y cells were lysed in buffer containing 50 mM Tris-HCl pH 8.0, 150 mM NaCl, 1% Triton X-100, 1 mM EDTA, 1 mM Na_3_VO_4 _and 1 complete protease inhibitor cocktail tablet (Roche Diagnostics)/10 ml buffer. The cells were incubated with lysis buffer for 10 minutes on ice before scraping the cells from the dishes. Cell lysates were centrifuged at 14 000 rpm for 15 minutes. The protein content in the supernatants was measured using Pierce^® ^660 nm Protein Assay kit. Samples, containing 20 μg protein, were resolved in 7% NuPAGE^®^Tris-Acetate gels (InVitrogen) using Tris-Acetate SDS running buffer (InVitrogen). The proteins were transferred to PVDF membranes using iBlot™ gel transfer stacks (InVitrogen). Membranes were blocked in PBS with 0.05% Tween 20 containing 3% non-fat dry milk for 1 hour at RT. Mouse β amyloid (6E10) monoclonal antibody (Signet laboratories) was diluted 1:1000 and the β-actin antibody (Sigma-Aldrich) 1:10 000 in 1% milk and incubation was carried out at 4°C over night. Horseradish-peroxidase (HRP) conjugated anti-mouse secondary antibody (Amersham Biosciences) was incubated 1 hour at RT in 1% milk at the dilution of 1:3000. Blots were developed using the Super Signal^® ^detection system (Pierce). Average density of the bands was measured in ChemiDoc™XRS (Bio-Rad) by using Quantity One software.

## Results

### APP expression and Aβ secretion from APPwt- and APPswe-transfected SH-SY5Y cells

**Figure 2:**
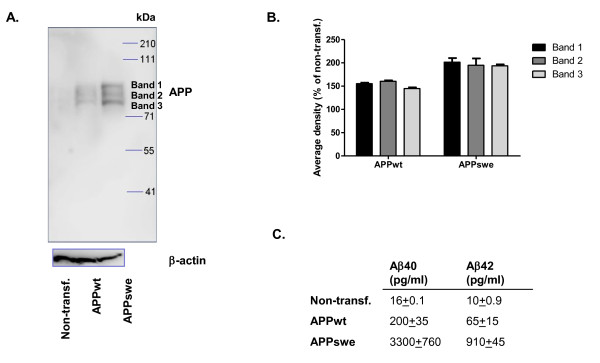
**APP expression and Aβ secretion in SH-SY5Y cells**. **A**. APP expression in non-transfected, APPwt- and APPswe-transfected SH-SY5Y cells. The three bands detected with 6E10 antibody in the cell lysates are most likely isoforms of full-length APP or mature/immature APP differentially modified by glycosylation. **B**. Densitometric quantification shows 50% increase of APP levels in APPwt transfected SH-SY5Y cells and 100% increase in APPswe transfected cells as compared to non-transfected ones. Data presented as means ± SEM, n = 2. **C**. Aβ40 and Aβ42 secretion during 24 h in non-transfected, APPwt- and APPswe-transfected SH-SY5Y cells.

Transfection of APPwt and especially APPswe genes into SH-SY5Y cells increased APP expression compared to non-transfected cells (Figure 2A). The 6E10 antibody that binds to amino acid residues 1-16 of Aβ recognized three bands that are most likely isoforms of full-length APP or mature/immature APP differentially modified by glycosylation. Densitometric quantification of Western blot is shown in Figure 2B. The increase in APP level is approximately 50% in APPwt transfected SH-SY5Y cells and 100% in APPswe transfected cells.

Baseline secretion of Aβ40 and Aβ42 during 24 h from non-transfected SH-SY5Y cells was only slightly above the level of detection while being readily measurable in cells transfected with APPwt (Figure 2C). By contrast, secreted Aβ40 and Aβ42 levels were approximately 15-fold higher from APPswe-transfected cells compared to APPwt-transfected (Figure 2C). APPswe gene has a double mutation at codons 670 and 671 located just N-terminal of the Aβ N-terminus, which makes APP a better substrate for BACE resulting in increased production of total Aβ[23].

### Effect of the γ-secretase inhibitor LY450139 on Aβ40 and Aβ42 secretion from SH-SY5Y APPwt and APPswe cells

**Figure 3:**
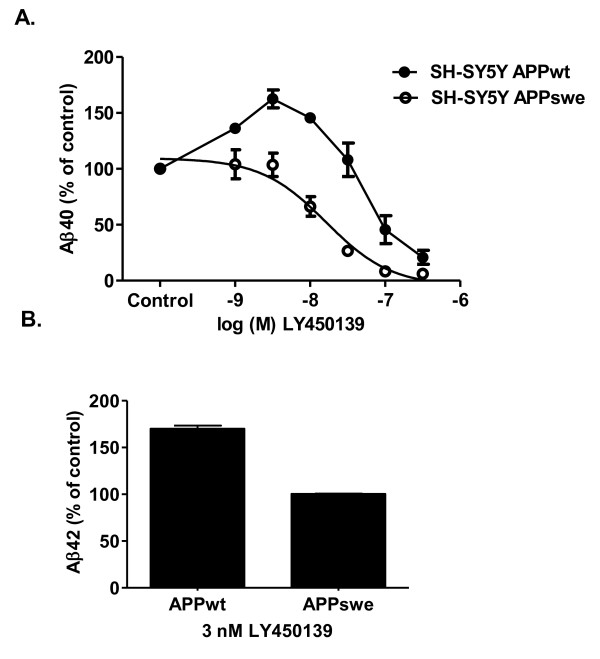
**Effect of LY450139 on Aβ secretion in SH-SY5Y cells**. **A**. In SH-SY5Y APPwt cells (closed circles), LY450139 produced a biphasic response with Aβ40-levels increasing in response to low concentrations of inhibitor, reaching 60% above baseline at 3 nM, while at higher concentrations Aβ40 levels started to decrease with an approximate IC_50 _of 90 nM. In APPswe-transfected SH-SY5Y cells (open circles), LY450139 produced a monophasic concentration-dependent inhibition of Aβ40-secretion with an IC_50 _of 18 nM. **B**. LY450139 (3 nM) increased Aβ42 levels by 70% as compared to control in SH-SY5Y APPwt cells, whereas Aβ42 levels in APPswe cells were not affected.

In SH-SY5Y APPwt cells, the γ-secretase inhibitor LY450139 produced a biphasic response with Aβ40 levels increasing in response to low concentrations of inhibitor, reaching 60% above baseline at 3 nM (Figure 3A). At higher concentrations (> 30 nM), Aβ40 levels decreased with an approximate IC_50 _of 90 nM. By contrast, in SH-SY5Y APPswe cells, LY450139 produced a concentration-dependent inhibition of Aβ40-secretion with a monophasic profile and an IC_50 _of 18 nM (Figure 3A). Hence, under our experimental conditions, LY450139 was ~5-fold less potent at inhibiting Aβ40-secretion from APPwt cells compared to APPswe cells.

At 3 nM, LY450139 also increased secreted Aβ42 levels by 70% in SH-SY5Y APPwt cells compared to controls, while secreted Aβ42 levels from APPswe cells were not affected (Figure 3B).

### Washout experiments with LY450139 in SH-SY5Y APPwt and APPswe cells

**Figure 4:**
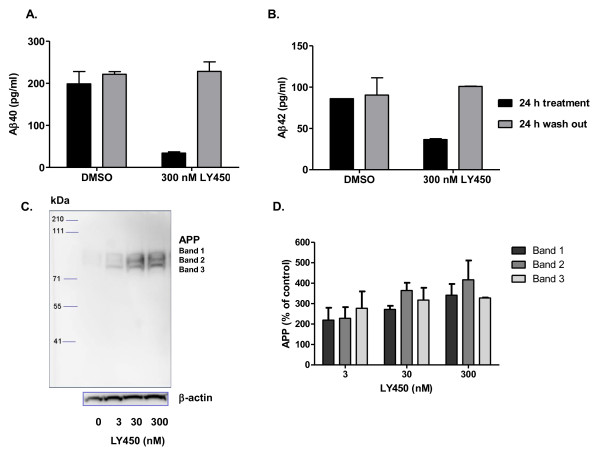
**Washout experiments with LY450139 in SH-SY5Y cells**. Inhibition with high concentration of γ-secretase inhibitor LY450139 leads to substrate accumulation and decreased Aβ secretion but no rebound is detected after washing out the inhibitor. SH-SY5Y APPwt cells were treated with vehicle (0.1% DMSO) or 300 nM LY450139 for 24 h, after which the medium was collected for Aβ40 (**A**) or Aβ42 (**B**) measurements (black bars). The media was replaced with fresh medium for another 24 h, and again collected for Aβ40 (**A**) or Aβ42 (**B**) measurements (grey bars). **C**. All three bands detected with 6E10 antibody increased in density in the cells treated with 3, 30 or 300 nM LY450139 indicating substrate accumulation. **D**. Densitometric quantification of data in C as means ± SEM, n = 2.

The previously mentioned experiments were performed under closed-conditions, i.e. LY450139 was present during the whole incubation period (24 h) at a presumed constant concentration. In order to detect a possible Aβ rebound, we performed washout experiments. Incubating SH-SY5Y APPwt cells with 300 nM LY450139 for 24 h reduced Aβ40 secretion by 80% compared to control (Figure 4A), consistent with previous results (see Figure 3A). After 24 h, The LY450139-containing media and control media were washed out and replaced with fresh media without inhibitor and Aβ40 secretion was followed for an additional 24 h. In control cells, Aβ40 secretion during the subsequent 24 h did not differ from Aβ40 secretion during the initial 24 h indicating that washout *per se *does not affect SH-SY5Y secretory function (Figure 4A). Aβ40 secretion during 24 h from SH-SY5Y APPwt cells that had been pre-treated with 300 nM LY450139-containing media did not differ from control cells.

In analogy to Aβ40, Aβ42 secretion from SH-SY5Y APPwt cells returned to control levels after inhibition with 300 nM LY450139 for 24 h without any evidence of a rebound (Figure 4B).

Treatment with 3, 30 or 300 nM LY450139 for 24 h resulted in accumulation of substrate as shown in Figure 4C with densitometric quantification of the bands summarized in Figure 4D. This suggests that the substrate accumulation seen during the initial 24 hours did not lead to excessive Aβ40 secretion during the next 24 h. Shorter time periods (2 - 6 hours) after washout were also monitored in order to detect any possible immediate and short-acting Aβ40 rebound. However, Aβ40 production did not differ between LY450139 pre-treated and control cells at these shorter time points (data not shown).

**Figure 5:**
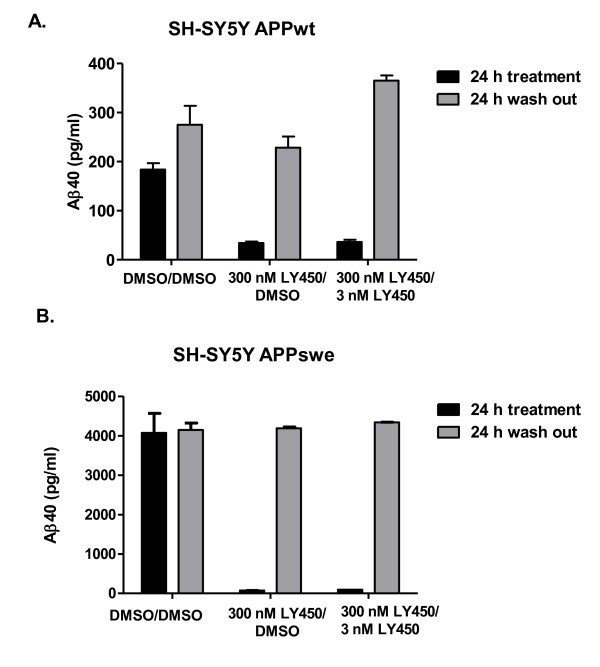
**Treatment with high concentration of γ-secretase inhibitor LY450139 followed by low concentration of LY450139 in SH-SY5Y cells**. Treatment with 3 nM LY450139 after inhibition by 300 nM LY450139 leads to increased Aβ40 secretion in SH-SY5Y APPwt cells but not in APPswe cells. SH-SY5Y APPwt cells (**A**) or APPswe cells (**B**) were treated with vehicle (0.1% DMSO) or 300 nM γ-secretase inhibitor LY450139 for 24 h, after which the medium was collected for Aβ40 measurements (black bars). The media was replaced with fresh medium (left panel and middle panel) or 3 nM LY450139 (right panel) for another 24 h, and then again collected for Aβ40 measurements (grey bars).

Since 3 nM LY450139 evoked an Aβ rise from SH-SY5Y APPwt cells under standard closed conditions (see Figure 3A), we wished to evaluate how this low concentration of inhibitor would act following inhibition of Aβ production over 24 h with a high concentration (300 nM) of LY450139 which leads to substrate accumulation. Treatment with 300 nM LY450139 over 24 h inhibited Aβ secretion by 80% as expected (Figure 5A). Replacing the medium with 3 nM LY450139 during the second 24 h incubation period, increased Aβ40 levels by 60% compared to the cells that during the first 24 h period received 300 nM LY450139 and during the second incubation period received fresh medium (Figure 5A). This phenomenon was not seen in SH-SY5Y APPswe cells, where treatment with 300 nM LY450139 followed by 3 nM LY450139 did not lead to increased Aβ40 levels (Figure 5B). Hence, 3 nM LY450139 evokes a similar degree of Aβ rise in SH-SY5Y APPwt cells, regardless if cells are pre-treated with a high concentration of inhibitor (leading to substrate accumulation) or not.

### Effect of concomitant BACE-1 inhibition on the Aβ rise evoked by LY450139

**Figure 6:**
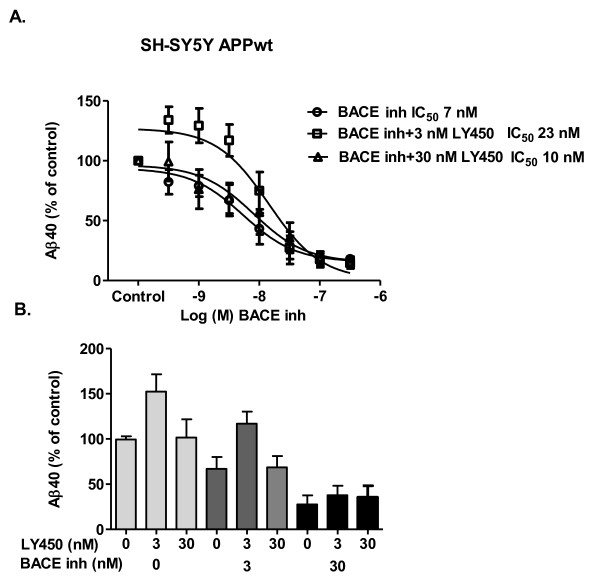
**Combining BACE- and γ-secretase inhibitor in SH-SY5Y APPwt cells**. **A**. SH-SY5Y APPwt cells were treated with the BACE-inhibitor alone or in combination with 3 or 30 nM γ-secretase inhibitor LY450139 for 24 h. Aβ40 levels were subsequently measured in the medium with ELISA. The BACE inhibitor produced a concentration-dependent inhibition of Aβ40-secretion with it's potency shifted to the right in the presence of 3 nM LY450139. **B**. The same data as in Figure 6A but depicted in a different manner. Here one can see how various concentrations of the BACE inhibitor (0: open bars, 3 nM: grey bars, 30 nM: black bars) affect the response to 0, 3 and 30 nM LY450139. The figure highlights that the LY450139-evoked Aβ40 rise is still present but attenuated in the presence of 3 nM of the BACE inhibitor. 30 nM BACE inhibitor is required to abolish the LY450139-evoked Aβ40 rise completely.

In SH-SY5Y APPwt cells, the BACE inhibitor inhibited Aβ40-secretion in a concentration-dependent manner with an IC_50 _of 7 nM and prevented the Aβ rise evoked by 3 nM LY450139 with an IC_50 _of 23 nM (Figure 6A). The BACE inhibitor had a similar potency when constructing the concentration response curve in the presence of 30 nM LY450139 (a concentration which *per se *does not affect basal Aβ40 levels) as without LY450139 (10 nM vs. 7 nM, Figure 6A). In Figure 6B the same results (from Figure (6A) are illustrated in an alternative manner. Here it is highlighted how the LY450139-evoked Aβ40 rise is still present but attenuated in the presence of 3 nM BACE inhibitor. 30 nM BACE inhibitor was required to abolish the LY450139-evoked Aβ40 rise completely.

**Figure 7:**
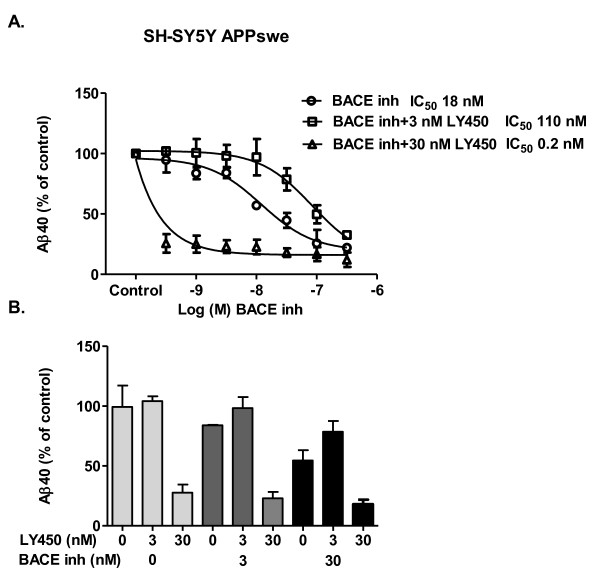
**Combining BACE- and γ-secretase inhibitor in SH-SY5Y APPswe cells**. **A**. SH-SY5Y APPswe cells were treated with the BACE-inhibitor alone or in combination with 3 or 30 nM γ-secretase inhibitor LY450139 for 24 h. Aβ40 levels were subsequently measured in the medium with ELISA. The BACE inhibitor produced a concentration-dependent inhibition of Aβ40-secretion with it's potency shifted to the right in the presence of 3 nM LY450139. At 30 nM, LY450139, together with the BACE inhibitor abolished Aβ40-secretion. **B**. The same data as in Figure 7A but depicted in a different manner. Here one can see how various concentrations of the BACE inhibitor (0: open bars, 3 nM: grey bars, 30 nM: black bars) affect the response to 0, 3 and 30 nM LY450139. The figure highlights that 3 nM LY450139 seems to oppose the inhibition evoked by the BACE inhibitor.

The same series of experiments were performed in SH-SY5Y APPswe cells. The BACE inhibitor reduced basal Aβ40-secretion from SH-SY5Y APPswe cells in a concentration-dependent fashion with an IC_50 _of 18 nM (Figure 7A). The potency of the BACE inhibitor was weaker (IC_50 _110 nM, Figure 7A) in the presence of 3 nM LY450139 (a concentration that does not affect Aβ40-secretion *per se *- see Figure 3A). The figure highlights that 3 nM LY450139, although not producing an Aβ40 rise in APPswe transfected cells, appears to counteract the potency of the BACE inhibitor. In the presence of 30 nM LY450139, the BACE inhibitor reduced remaining Aβ40 secretion (20-30% remaining) with an IC_50 _of 0.2 nM (Figure 7A). Figure 7B illustrates the data in a different manner. The figure highlights that overall Aβ40 levels are reduced when adding increasing concentrations of BACE inhibitor, although it seems as if 3 nM LY450139 under these conditions tends to increase Aβ40 levels compared to control cells treated with BACE inhibitor alone, reminiscent of the Aβ rise seen in APPwt cells.

## Discussion

Attenuating Aβ production, for instance by inhibiting either of the respective proteases BACE-1 or γ-secretase, is considered an attractive strategy for preventing disease progression in patients suffering from Alzheimer's Disease. However, both of these protease inhibition approaches have met several challenges over recent years. BACE-1 has been a difficult target from a chemical tractability point of view with few compounds entering clinical development, most likely due to the difficulties in achieving the combination of necessary enzyme inhibition with adequate brain exposure. Moreover, compounds that target γ-secretase have been associated with severe side effects since several other substrates with likely physiological relevance are cleaved by the γ-secretase complex. When compound exposure wanes, treatment with γ-secretase inhibitors actually results in increased Aβ levels, a so called Aβ rebound/rise. Considering these facts we felt it important to investigate whether combining a BACE inhibitor with a γ-secretase inhibitor would result in synergistic efficacy and whether a BACE inhibitor could prevent the Aβ rebound/rise evoked by a γ-secretase inhibitor.

In our first set of experiments, we were able to verify that LY450139 increases both Aβ40 and Aβ42 levels at a low concentration in SH-SY5Y APPwt cells but not in SH-SY5Y APPswe cells. At higher concentrations, inhibition occurred with LY450139 being 5-fold less potent at inhibiting Aβ secretion when using APPwt cells compared to APPswe cells. The potency of LY450139 using APPwt cells was similar to data reported from others [10] and the 5-fold shift in potency was very similar to Burton et al. [12], although they studied the γ-secretase inhibitor DAPT. This potency shift is presumably due to the differences in substrate/enzyme ratios.

The Aβ rise/rebound has been claimed to involve substrate accumulation due to inhibition of the γ-secretase complex. The rationale being that after inhibition has subsided the accumulated substrate, BACE-1 cleaved fragment C99, can more readily be converted to Aβ resulting in the Aβ rebound. However, sub-inhibitory doses of γ-secretase inhibitor also appear to increase Aβ levels suggesting instead that an Aβ rise occurs at low concentrations without previous inhibition [12]. Since the aim of this paper was to examine the effect of combining a γ-secretase inhibitor with a BACE inhibitor, we felt it important to first further elucidate whether a rebound-like effect or an Aβ rise was occurring in response to γ-secretase inhibition before addressing the combination of inhibitors.

If an Aβ rebound mechanism was behind the increased Aβ levels seen in our studies *in vitro*, then one would expect to see increased Aβ levels in the cell medium after first exposing cells to a high concentration of compound leading to γ-secretase inhibiton followed by washout. We tested this hypothesis in SH-SY5Y APPwt cells. Despite obvious substrate accumulation and decreased Aβ secretion in response to γ-secretase inhibition, subsequent over-production of Aβ was not detected when the inhibitor was washed out. We checked even shorter time points in case over-production would have occurred in a transient manner but this was not the case.

Burton et al. [12] suggested that the Aβ rise would only occur under conditions where γ-secretase substrate levels are relatively low (i.e. SH-SY5Y APPwt cells). We tested this hypothesis by first giving an inhibitory concentration of LY450139 (300 nM) which resulted in substrate accumulation and thus higher substrate levels in APPwt cells and then replaced the medium with a low concentration of LY450139 (3 nM) which normally gives an Aβ rise in low-substrate conditions. However, despite the increased substrate levels, 3 nM LY450139 resulted in a similar Aβ rise as under control conditions without substrate accumulation. This could perhaps be due to the level of substrate not reaching levels present in for instance APPswe cells. Indeed, we were not able to detect an Aβ rise in APPswe cells consistent with previous reports [12]. Alternatively, the level of substrate does not affect the Aβ rise and other mechanisms are involved.

The results from the current paper suggest that γ-secretase inhibitors like LY450139 actually increase Aβ levels at low concentrations *in vitro *without the need of prior inhibition or substrate accumulation occurring. This phenomenon is thus better referred to as an Aβ rise taking place at low concentrations as also suggested by others [9,12].

Having established the mode of action of LY450139 *in vitro *(although not the detailed molecular mechanism), we next studied how a BACE inhibitor affects the LY450139-evoked Aβ rise and LY450139 inhibitory potency in SH-SY5Y APPwt and APPswe cells. The BACE inhibitor prevented the Aβ rise in a concentration-dependent manner in APPwt cells, although the BACE inhibitor potency was shifted to the right (from 7 nM to 23 nM) in the presence of 3 nM LY450139, the concentration that normally increases Aβ secretion. The concentration-dependent inhibition suggests that ongoing BACE-1 activity is required for the Aβ rise to occur. This offers further support that the γ-secretase inhibitor-induced increases in Aβ levels are not due to rebound effects in response to substrate accumulation (i.e. C99 fragment) since this mechanism would not likely be BACE-1 dependent. Combining a BACE inhibitor with 30 nM LY450139 did not have any obvious advantage in APPwt cells, the BACE inhibitor potency being more or less the same as in the presence of BACE inhibitor alone (10 nM vs.7 nM). LY450139 at the concentration of 30 nM does not *per se *affect secreted Aβ.

Interestingly, despite the lack of an Aβ rise in SH-SY5Y APPswe cells, 3 nM of LY450139 clearly shifted the BACE inhibitor concentration-response curve to the right (18 nM vs. 110 nM). This suggests that also in APPswe cells, a low concentration of LY450139 is trying to raise Aβ levels but not enough to detect under basal conditions. However, it appears to be manifested in the presence of the BACE inhibitor, not by increasing Aβ levels but by counteracting BACE inhibition. Indeed, small signs of an Aβ rise in response to 3 nM LY450139 can be seen in the presence of 3 and 30 nM BACE inhibitor in APPswe cells. It is possible that as γ-secretase substrate falls in response to BACE inhibition, an Aβ40 rise is triggered in response to low concentrations of LY450139 even in APPswe cells. By contrast, higher concentrations of LY450139 (30 nM), shifted the BACE inhibitor curve to the left, but it is important to keep in mind that this concentration of LY450139 *per se *has considerable inhibitory effects making conclusions on possible synergy difficult. The current study thus fails to detect any obvious synergies between γ-secretase and BACE-1 inhibition in SH-SY5Y APPwt- or in APPswe-transfected cells. However, closer titration of inhibitors is warranted in future studies, for instance using a higher concentration of LY450139 (e.g. 60 nM).

## Conclusions

Combined BACE-1 and γ-secretase inhibition is complex and the outcome is likely to vary depending on substrate levels. When viewing the results from γ-secretase perspective, BACE-1 inhibition can prevent the Aβ rise evoked by γ-secretase inhibition at low concentrations without showing any obvious potentiation at higher concentrations. By contrast, from a BACE-1 perspective, adding the γ-secretase inhibitor LY450139 did not potentiate BACE-mediated inhibition but rather shifted the concentration-response curve to the right, most likely due to LY450139 trying to raise Aβ levels at low concentrations. Hence, adding a BACE inhibitor to a γ-secretase inhibitor like LY450139 could have advantages by preventing the Aβ rise.

## Competing interests

The authors declare that they have no competing interests.

## Authors' contributions

AJ carried out the experiments in SH-SY5Y cells, participated in the design of the study and drafted the manuscript. OB was responsible for the synthesis of the inhibitors used in the study. ME designed the plasmids used for transfection of the cells. EL organized the study, participated in the study design and revised the manuscript. All authors read and approved the final manuscript.
